# Correlation analysis of hearing thresholds, validated questionnaires and psychoacoustic measurements in tinnitus patients

**DOI:** 10.1590/S1808-86942010000400018

**Published:** 2015-10-19

**Authors:** Ricardo Rodrigues Figueiredo, Marcelo A. Rates, Andréia Aparecida de Azevedo, Patrícia Mello de Oliveira, Patrícia B.A. de Navarro

**Affiliations:** a Msc in General Surgery-Otorhinolaryngology - Federal University of Rio de Janeiro, Full Professor of Otorhinolaryngology - Medical School of Valença, RJ. TeTechnical Diretor - OTOSUL, ENT Sul-Fluminense, Volta Redonda, RJ; b ENT, Assistant Professor of Otorhinolaryngology - Federal University of Minas Gerais, Belo Horizonte, MG. ENT physician - Centro de Tratamento e Pesquisa em Zumbido, Belo Horizonte, MG; c ENT Physician, Head of the OTOSUL Scientfc work - ENT Sul-Fluminense, Volta Redonda, RJ; d Speech and Hearing Therapist - Head of the Audiology Department - OTOSUL, ENT Sul-Fluminense, Volta Redonda, RJ; e Speech and Hearing Therapist - Tinnitus Research and Trainning Center - Belo Horizonte, MG

**Keywords:** audiometry, psychoacoustics, questionnaires, tinnitus

## Abstract

One of the most criticized points in tinnitus clinical studies arise from the lack of consensus about measurement methods.

**Aim:**

To evaluate the correlation between audiometric thresholds, pitch matching (PM), minimum masking level (MML), Tinnitus Handicap Inventory (THI) and the Beck Depression Inventory (BDI) in tinnitus patients.

**Study design:**

Prospective, cross-sectional.

**Materials and methods:**

Subjects were submitted to tonal audiometry, PM and MML for tinnitus. They also filled out the THI and BDI. Data was statistically compared for correlation purposes between audiometric thresholds, psycho-acoustic measures and questionnaires.

**Results:**

There was no statistically significant correlation between THI and MML, both in patients with BDI scores under and over 14 points. There was no statistically significant correlation between the worst hearing frequency and PM, as well as between the cut-off frequency and the PM in patients with descending hearing curves in their audiograms.

**Conclusions:**

There is no statistically significant correlation between psycho-acoustic measures (PM and MML), audiometric thresholds, THI and BDI. Tinnitus is a very complex symptom and isolated measures by psycho-acoustic methods; tinnitus and depression questionnaires are not satisfactory.

## INTRODUCTION

Tinnitus can be defined as an auditory sensation which is perceived by the patient, without any external physical source generating the sound. North American data estimates that between 15 and 20% of the population in general have tinnitus and in 20% of these individuals it has a major impact on their quality of life^1^. Results from the different tinnitus treatment strategies are rather inconsistent. Nonetheless, since some patients seem to benefit strongly from specific treatment modes, the current trend is that this symptom has a multifactorial origin with different subtypes^2^. One fact which makes the assessment of tinnitus patient particularly difficult is the lack of uniformity concerning its methods of investigation. Numerous publications have been criticized because of its methodology, especially concerning the tinnitus measuring and evaluation technique^3^. Meikle et al., in a review article from 2008^4^, classified these methods into 4 categories: psychoacoustic tests, scales, questionnaires to assess tinnitus functional effects and questionnaires to analyze the global perceptions of the therapeutic effects.

Psychoacoustic tests have been employed since the 40s4 and the four most used ones are: Pitch Match (PM), Loudness Match (LM), Minimum masking level (MML) and Residual Inhibition, (RI). The questionnaires used to assess the functional effects are made up of numerous items which assess tinnitus impact on numerous aspects of daily life^4^. According to some authors, its use guarantees greater reliability in the assessment of tinnitus when compared to other methods^5^. Nonetheless, for other authors, questionnaires for tinnitus assessment are not entirely reliable, since most of them were not developed with the aim of assessing treatment results4. Among the most used questionnaires, we list: Tinnitus Handicap Inventory (THI)^6^, Tinnitus Handicap Questionnaire (THQ)^7^ and the Tinnitus Questionnaire (Mini-TQ)^8^. The first two are commonly used in English speaking countries, and the third is used in Europe. In Brazil, the Brazilian-validated version of the THI^9^ is the one most used, as well as the visual-analogue scale^3^.

Tinnitus can be a significant problem in the lives of patients^2^^,^^3^ - bringing about great difficulty in their capacity to adapt to this new reality. Moreover, tinnitus is, in most of the cases, associated to hearing loss, which causes a significant additional impact. Therefore, it is greatly important to assess and, whenever possible, to measure the importance of the participation of the so-called “psychological aspects “ as worsening factors for the consequences brought about by tinnitus and its eventual impact on treatment results.

We know that depression is a mood disorder characterized by clinical aspects such as fatigue, reduction in concentration and attention, low self-esteem and self-confidence^10^. Depression can be classified in different levels: mild, moderate and severe, in function of quantity, type and intensity of symptoms^10^. Dysphonia is also a characteristic of many psychiatric disorders, including anxiety and mood disorders. It is usually characterized as an unpleasant sensation or annoyance such as sadness, anxiety, irritability or agitation. Etymologically it is the opposite of euphoria^10^. The Beck Depression Inventory (BDI)^11^ is probably the most used self-assessment method to study depression, both in clinical practice as in research^12^, having been translated and validated in numerous countries, including Brazil^12^.

Parallel to that, experimental studies in animals have shown that cochlear lesions can induce neural plasticity mechanisms both at a cortical and subcortical levels, followed by tonotopic reorganization, similarly to what happens in the amputation of limbs^13^^,^^14^. Cortical neurons, without afferent stimuli coming from the cochlea can suffer neuroplastic changes, with subsequent reduction in their firing thresholds for the frequencies around the affected areas, a theory known as “lesion-edge”^15^. Similar mechanisms can be associated to some forms of tinnitus^2^. Moreover, clinical observations have led to the assumption that the cochlear damage, similarly to what happens in the training of perceptions, is followed by a better performance of auditory discrimination in specific frequencies^16^. Clinical studies in humans reinforce these concepts, showing through the Frequency Difference Limens (FDL) in patients with sensorineural hearing loss and descending audiometric curves, that small differences are found in those frequencies near the cut-off frequencies^16^.

With this study we aim at assessing the correlation between audiometric data, psychoacoustic measures associated to tinnitus (TM and MML) and questionnaires validated for Brazilian Portuguese (THI and BDI).

## MATERIALS AND METHODS

Forty-eight (48) patients with tinnitus as chief complaint, who came for treatment in the referral centers were selected, 30 coming from Center A and 18 from Center B. We took off the study those patients younger than 18 years of age, pregnant women, patients with tinnitus for less than 3 months; tinnitus of vascular, muscular or somatosensory origin; patients with TMJ disorders, those with changes seen upon otoscopy; conductive or mixed hearing loss; patients with tympanic curves A-r, A-d, B and C, patients submitted to medication treatment for tinnitus in the past 6 months and those with VIII nerve schwannoma (MRI carried out before inclusion in the study in the cases of asymmetric dysacousia).

All the patients were submitted to tonal audiometry, immittance test, Pitch Match (PM) - a test in which the patient correlates tinnitus with a narrow band or pure tone noise, Minimum masking level (MML) - a test in which the patient is exposed to a broad band noise, which is adjusted at each 1-2 dB, until the patient reports no longer perceiving the tinnitus; and the Cut-off frequency - Fc, defined as the greatest frequency at which the auditory threshold is up to 5 dB above the best threshold, measured only on the descending audiometric curves^17^. The methodology followed was exactly the same in both centers.

All the patients also answered the THI (Tinnitus Handicap Inventory) and BDI (Beck Depression Inventory) questionnaires, both in their validated versions for Brazilian Portuguese^9^^,^^12^.

The parameters evaluated for the sample set were: gender, age, laterality and tinnitus type and duration. The statistical data was obtained in order to establish possible correlations between MML and THI (with the goal of assessing the correlation between the intensity with which the patient perceives the tinnitus and the loss caused by it), between THI, MML and BDI (with the goal of checking whether the correlation between THI and MML can suffer any interference from depression), among the frequencies with the worst auditory thresholds and PM; and between Fc and PM (the latter aim at checking whether there is audiometric evidence which corroborates the lesion-edge theory - in tinnitus patients).

In order to compare MML-PM and the categorical variables of gender, laterality, THI and BDI we carried out the t-student test with habitual assumptions of the model (normality and homoscedasticity) and 2 groups were compared, otherwise the Mann-Whitney test was employed. The hypothesis of the t-student test was checked with the Kolmogorov-Smirnov test for the normality and with the Levene test for homoscedasticity. In the cases in which more than 2 categories were compared, the F test was done when the hypothesis was confirmed, otherwise the Kruskal-Wallis test was used. In order to compare MML-PM and the quantitative parameters of age, tinnitus time, THI, BDI, frequencies of the worst hearing threshold and the cut-off frequencies, the Pearson correlation coefficient was used.

The study was approved by the Ethics in Research Committee, resolution number 005/2009, and recorded at the ClinicalTrials.gov under # NCT 00976547.

## RESULTS

The demographic characteristics, qualitative and quantitative data for the entire sample (n=48) are detailed on Table 1, Table 2.

**Table 1:** Categorical variables for the total sample (n=48).

Covariable	Frequency	
	n	%
Gender		
Male	23	47,9
Female	25	52,1
Laterality		
Right ear	8	16,7
Left ear	12	25,0
Both ears	25	52,0
CEF (tinnitus perceived in the head)	3	6,3
Worse tinnitus side considering both ears		
Right ear	6	24,0
Left ear	7	28,0
Without identification	12	48,0
Worst auditory threshold in the right ear in		
1 frequency	34	70,8
2 frequencies	11	22,9
3 frequencies	2	4,2
4 frequencies	1	2,1
Worst auditory threshold in the left ear in		
1 frequency	36	75,0
2 frequencies	9	18,7
3 frequencies	2	4,2
Deafness	1	2,1
THI		
0 to 16 mild tinnitus (Degree 1)	6	12,5
18 to 36 mild tinnitus (Degree 2)	12	25,0
38 to 56 moderate tinnitus (Degree 3)	16	33,3
58 to 76 Severe tinnitus (Degree 4)	9	18,8
78 to 100 catastrophic tinnitus (Degree 5)	5	10,4
BDI		
Below 14	39	81,3
15 to 20 - Dysphonia	5	10,4
Above de 21 - Depression	4	8,3

**Table 2:** Quantitative variables for the entire sample (n=48).

Covariable	n	n*	Mean	SD	Minimum	Maximum	Median
Age (years)	48	0	56,3	12,5	22,0	80,0	56,5
Evolution time (years)	48	0	8,5	8,8	1,0	38,0	5,5
MML right ear (dB)	36	12	50,4	21,2	9,0	95,0	51,0
MML left ear (dB)	40	8	50,8	18,7	15,0	93,0	50,0
TM right ear (Hz)	36	12	5.499,0	3.018,0	250,0	11.200,0	6.000,0
TM left ear (Hz)	40	8	5.555,0	2.966,0	500,0	11.200,0	6.000,0
THI	48	0	45,5	24,0	4,0	98,0	46,0
BDI	48	0	9,4	7,4	0,0	29,0	9,0

According to the Mann-Whitney, t Student, Kruskal-Wallis and F Tests, there was not statistical correlation between MML and the variables: gender and laterality, as well as THI and BDI scores. The Pearson's Correlation Coefficient is depicted on Table 3, with statistical tendency towards higher MML values being perceived by elderly patients with unilateral hypoacusis (p- value of around 0.05).

**Table 3:** Pearson's Correlation Coefficient in patients with unilateral disacousia.

Covariable	r	p-value
Age (years)	0,409	0,074
Evolution time (years)	0,062	0,795
THI	0,303	0,195
BDI	0,084	0,724

Moreover, we did not see statistically significant differences (Pearson's Coefficient) on the MML-THI correlation between the subgroups with normal and abnormal BDI scores, as detailed on Table 4.

**Table 4:** Pearson's Correlation Coefficient between MML and THI among patients with normal BDI (< or equal to 14) and abnormal (>14).

	R	p-value
Normal BDI		
Right ear MML and THI	0,286	0,133
Left ear MML and THI	0,034	0,855
Abnormal BDI		
Right ear MML and THI	0,503	0,250
Left ear MML and THI	0,463	0,248

According to Mann-Whitney, Pearson, t Student, Kruskal-Wallis and F tests, we did not find statistical correlations between PM and the variables: gender, age and laterality, as well as the THI and BDI scores. We also did not notice statistical correlations between PM and the audiometric data, including the worst threshold and cut-off frequency (p > 0.05), the latter was analyzed in 30 patients with descending audiometric curves.

## DISCUSSION

Our results are in accordance with those from Hiller & Goebel's study, in which they analyzed the correlation between the Mini-TQ questionnaire and the Klochoff & Lindblom loudness grading system in 4,958 tinnitus patients (retrospective study)^18^. The authors concluded that the volume of the tinnitus perceived by the patient and the annoyance are not necessarily in agreement; and therefore must be assessed separately. On the other hand, Meikle found a positive correlation between the questionnaire used to assess tinnitus and the subjective volume perceived by the patient^19^.

The incidence of depression in our sample was low (8.3%), in agreement with studies led by Newman et al., in which we noticed a weak correlation between tinnitus and depression^6^. However, other studies have established a clear correlation between tinnitus and depression^7^^,^^20^. One explanation for such fact may reside in the mean THI of our sample (45.5 - moderate score). We agree with Newman, since, in our opinion this population represents more reliably the tinnitus patients regularly seen by ENTs. Many clinical studies in tinnitus patients have in their samples high mean THI values, which are frequently associated with depression. Such fact can be associated with different results from different treatment modalities.

Our data shows a mild statistical trend towards higher MML in elderly patients with unilateral dysacousia, and such finding can be interpreted carefully because of its little statistical representativeness.

An interesting finding was the high MML in the sample (mean of 50.6 dB) in comparison to other studies (mean of 15 dB)^21^. Since the methodology of audiologic tests was similar to that from other studies a possible explanation could be associated to the presence of different subtypes of tinnitus or to variations associated with the patient's skill to perceive and compare sounds (sociocultural aspects).

Our data are in disagreement with some studies which established a correlation between the tinnitus frequency and the hearing loss frequencies^22^^,^^23^. Moreover, there was no correlation between Fc and PM. Considering the fact that about 90% of the patients with tinnitus have hearing loss1 and the most current theories associate tinnitus with central neuroplastic changes which follow an initial damage - usually cochlear^2^, it is reasonable to assume eventual correlations between the hearing loss frequencies (or neighboring to them) and the tinnitus frequency. In our study we were unable to establish such relations given the relatively small number of patients. The data set reinforces the extreme complexity of tinnitus as a symptom, not being restricted to the interactions between sensorineural hearing loss, neuroplasticity and, eventually, depression.

Our data reinforces the extreme complexity of tinnitus as a symptom. We believe that a standardized assessment strategy could be extremely useful in the standardization and reproducibility of clinical studies about tinnitus, since there is no correlation between numerous methods currently in use.

## CONCLUSION

We did not notice statistically significant correlations between psychoacoustic measures (Pitch matching and Minimum Masking Level), audiometric data (frequency with the worst threshold and cut-off frequencies) and questionnaires validated to assess tinnitus (Tinnitus Handicap Inventory) and depression (Beck Depression Inventory).
